# Is previous influenza-like illness a potential Trojan horse for COVID-19?

**DOI:** 10.1186/s13054-020-03226-5

**Published:** 2020-08-14

**Authors:** Giancarlo Ceccarelli, Gabriele d’Ettorre, Giuseppe Pietro Innocenti, Claudio M. Mastroianni, Massimo Ciccozzi, Gabriella d’Ettorre

**Affiliations:** 1 Department of Public Health and Infectious Diseases, Sapienza University of Rome, Viale del Policlinico 155, 00161 Rome, Italy; 2 Azienda Ospedaliero-Universitaria Policlinico Umberto I, Rome, Italy; 3 Unit of Occupational Prevention and Protection, ASL Lecce, Lecce, Italy; 4 Department of Medical Statistics and Epidemiology Unit, Campus Bio-Medico University, Rome, Italy

Dear Editor,

We read with interest the paper by Verroken et al. underlining that no respiratory virus coinfections were identified in a population of COVID-19 critically ill patients [1]. A recent large cohort study by Nowak et al. confirmed that SARS-CoV-2 and respiratory virus coinfections are rare events observed only in less than 3%, despite the temporal overlap of the two epidemic curves. Interestingly, they observed that 13% of COVID-19-free patients had isolation from the respiratory secretions of influenza virus, rhinovirus/enterovirus, and coronavirus NL63, in the same period [2]. Although with wide geographical variability, this data is in line with the epidemiology of respiratory viral pathogens in the northern hemisphere during the winter period [3], while deviates from the expected in SARS-CoV-2 infected patients, suggesting a possible viral interference [2].

Otherwise, Italy was affected by the SARS-CoV-2 outbreak since mid-February 2020, immediately after the period in which the incidence of influenza-like illness (ILI) gradually increased until reaching the epidemic peak in the fifth week of 2020, with a level equal to about 13 cases per thousand assisted [4]. The complete epidemiological data from 2019/2020 influenza season are not yet available, but we can assume that the aetiology of ILI was similar to that reported for the 2018/2019, when about 8 million of cases were registered in Italy and 31.7% caused by influenza viruses [4]. In accordance with epidemiological data, we also observed that 63.6% of 190 COVID-19 patients (admitted to Policlinico Umberto I Hospital of Rome, one of the larger teaching Italian COVID-19 Medical Centers in March 2020) reported in their clinical history a recent ILI (1–3 weeks prior to the appearance of COVID-19-related symptoms). Previous flu vaccination was reported in only 26.3% of patients. The symptomatology reported for the ILI was mainly characterised by sore throat, cough, runny nose, and conjunctivitis. These findings, although not conclusive, seem to suggest that ILI may represent a risk factor for a subsequent SARS-CoV-2 infection. In confirmation of this, interestingly, a number of ILI-related viral pathogens (i.e. *respiratory syncytial virus* and influenza virus) have been reported to cause a significant downregulation of ACE2 in the upper and lower respiratory tract, since the early stage after the onset of infection [5]. The consequent reduction of ACE2 activity has been found potentially contributing to severe lung injury and may predispose to a later more severe clinical course of COVID-19 [6]. Moreover, considering that intercurrent viral respiratory infections are a trigger of upper airway mucosal damage and local immune impairment, previous ILI could therefore represent a predisposing factor for subsequent COVID-19 infection. On the basis of these data, influenza vaccine not only has a public health utility permitting to exclude influenza in patients with ILI during the overlapping of the two epidemic curves, but probably also reduces the risk and the severity of COVID-19. Nevertheless, more than 65% of ILI have a non-flu aetiology; for these reasons, the implementation of behavioural containment measures is needed to reduce the risk of ILI spreads in areas affected by the SARS-CoV-2 outbreak.

## Data Availability

On demand to corresponding author
